# Assessment of cup orientation in hip resurfacing: a comparison of TraumaCad and computed tomography

**DOI:** 10.1186/1749-799X-8-8

**Published:** 2013-04-11

**Authors:** Daniel J Westacott, John McArthur, Richard J King, Pedro Foguet

**Affiliations:** 1 Warwick Orthopaedics, University Hospital of Coventry and Warwickshire, Coventry, UK

**Keywords:** Hip, Resurfacing, Orientation, Version, Software

## Abstract

**Purpose:**

The orientation of the acetabular component in metal-on-metal hip resurfacing arthroplasty affects wear rate and hence failure. This study aimed to establish if interpretation of pelvic radiographs with TraumaCad software can provide a reliable alternative to CT in measuring the acetabular inclination and version.

**Methods:**

TraumaCad was used to measure the acetabular orientation on AP pelvis radiographs of 14 painful hip resurfacings. Four orthopaedic surgeons performed each measurement twice. These were compared with measurements taken from CT reformats. The correlation between TraumaCad and CT was calculated, as was the intra- and inter-observer reliability of TraumaCad.

**Results:**

There is strong correlation between the two techniques for the measurement of inclination and version (p <0.001). Intra- and inter-observer reliability of TraumaCad measurements are good (p <0.001). Mean absolute error for measurement of inclination was 2.1°. TraumaCad underestimated version compared to CT in 93% of cases, by 12.6 degrees on average.

**Conclusions:**

When assessing acetabular orientation in hip resurfacing, the orthopaedic surgeon may use TraumaCad in the knowledge that it correlates well with CT and has good intra- and inter-observer reliability but underestimates version by 12° on average.

## Introduction

A high inclination angle of the acetabular component in hip resurfacing is associated with high serum metal ion levels and pseudotumour formation due to edge-loading and increased wear rate [1-4]. Recent studies have suggested that excessive anteversion may be an equally important factor [4-6] but these findings have not been consistently reproduced across the literature [1,7]. This difference may be due to the difficulties in accurately measuring anteversion.

Incorrect version can also cause iliopsoas irritation [8] and impingement, reducing range of motion and increasing the risk of fracture, dislocation and loosening [9,10]. Accurate assessment of acetabular orientation is therefore key in evaluating a painful hip resurfacing.

Radiological anteversion was described by Murray as the angle between the axis of the acetabulum and the coronal plane [11]. Various techniques exist for measuring cup anteversion. Cross-table lateral radiographs are often used but have been shown to be of limited use [12]. Evaluation of antero-posterior radiographs with EBRA (Einzel-Bild-Roentgen-Analysis, University of Innsbruck, Austria) has been validated as an accurate method in hip resurfacing [13] but this software is not widely available in UK centres. TraumaCad (Voyant Health, Petach-Tikva, Israel) also offers a measurement tool for cup version (and inclination) but it has not been validated in the peer-reviewed literature. Unlike EBRA, TraumaCad does not allow users to input bony landmarks to take into account the position of the pelvis, although it uses the same elliptical principle, first described by McLaren [14] and modified by Widmer [15]. Computed tomography (CT) has been widely used to measure cup orientation [2,12,16-18] and shown to be more accurate than manually-interpreted plain radiographs in hip resurfacings [19] but involves significant radiation exposure.

We therefore sought to determine if analysis of pelvic radiographs with TraumaCad software is a reliable method of assessing acetabular component orientation in hip resurfacing by assessing the correlation between TraumaCad and CT, and the intra- and inter-observer reliability of TraumaCad.

## Methods

**Figure 1:**
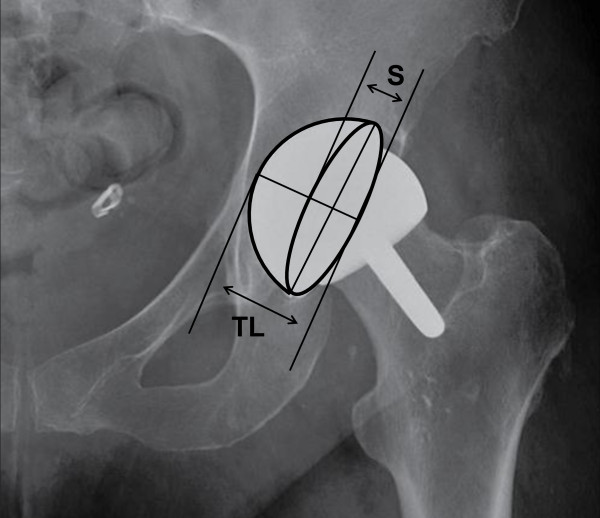
Short axis (S) of projected ellipse and total length (TL) of projected cup cross-section provide the S/TL ratio from which the version is calculated.

14 hips in 12 patients with symptoms of groin pain following arthroplasty with the Cormet hip resurfacing system (Corin, Cirencester, UK) were investigated with an antero-posterior pelvic radiograph and CT as part of their routine clinical assessment. Patient data were anonymised to all investigators. Radiographs were taken with the patient supine and the legs slightly abducted and internally rotated so the feet made a right angle with the toes touching. The beam source was 100 cm from the receiver and at 90° to the table. Four orthopaedic surgeons (two Consultants and two Specialist Registrars), who were familiar with the software, used TraumaCad to measure the version and inclination of each acetabular component. The technique calculates the version by comparing the size of the ellipse created by the oblique view of the articular surface of the component (S) with the total diameter of the cup’s cross-sectional projection (TL), demonstrated in Figure 1. The radiological inclination is the angle between the long axis of the ellipse and the intertuberous line. The process was repeated by the same four surgeons one week later, assessing the radiographs in a randomly different order.

**Figure 2:**
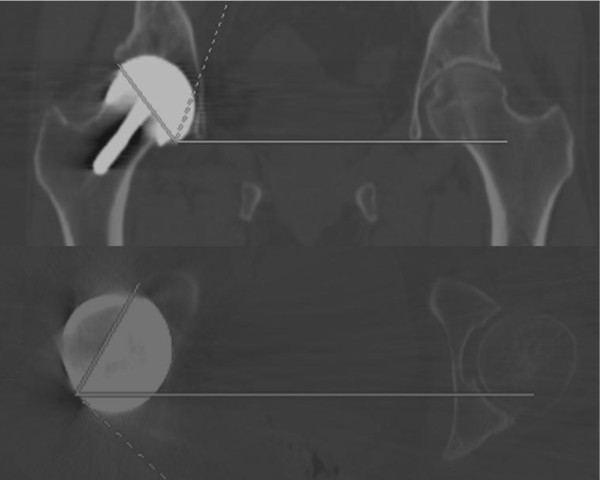
Technique for measuring version and inclination of the acetabular component using CT reconstructions.

Pelvic CT scans with 0.625 mm slice thickness were interpreted by an experienced musculoskeletal radiologist using ADW light reformat software (GE Healthcare Europe GmbH, Munich, Germany). To measure the inclination, a coronal plane was first defined by referencing from the posterior columns on an axial section. Having translated this plane to the apex of the cup, the inclination was measured as the angle made between the cup apex and a line parallel to the inferior aspects of the tear drops. To measure the version, a true trans-axial plane was defined at 90° to the coronal plane at the level of the cup apex and the angle measured between the cup apex and the trans-pelvic plane. The technique is demonstrated in Figure 2. The radiologist was not blinded to patient history or symptoms.

Pearson’s correlation coefficient was used to assess the correlation between TraumaCad and CT measurements for inclination and version, while the mean absolute error between the measurements of the two methods was calculated. Intra- and inter-observer reliability was calculated using the intra-class correlation co-efficient. Statistical analysis was performed with SPSS 17.0 (IBM, Armonk, NY).

Ethical approval was not required in accordance with United Kingdom National Research Ethics Service guidelines regarding the use of anonymised patient data collected as part of routine clinical care.

## Results

All the acetabular components were in anteversion on CT (mean 23.4°, range 1° to 44°). Mean inclination on CT was 49.3° (range 37° to 64°).

**Figure 3:**
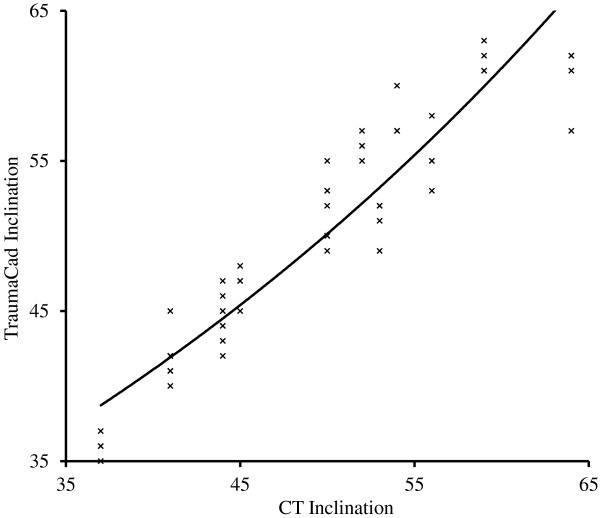
**Relationship between measurements of inclination from CT and TraumaCad.** The data plots represent each observer’s first measurements with a line of best fit.

There was strong correlation between TraumaCad and CT for the measurement of inclination (Table 1). The mean absolute error was 2.1°. Figure 3 shows the equal relationship between the two methods.

**Table 1:** Correlation between CT and TraumaCad

		**Observer 1**		**Observer 2**		**Observer 3**		**Observer 4**	
		**1**^**st **^**reading**	**2**^**nd **^**reading**	**1**^**st **^**reading**	**2**^**nd **^**reading**	**1**^**st **^**reading**	**2**^**nd **^**reading**	**1**^**st **^**reading**	**2**^**nd **^**reading**
Inclination	PCC*	0.904	0.917	0.949	0.961	0.969	0.953	0.962	0.947
	*p* value	<0.001	<0.001	<0.001	<0.001	<0.001	<0.001	<0.001	<0.001
Version	PCC*	0.909	0.865	0.892	0.907	0.886	0.891	0.880	0.913
	*p* value	<0.001	<0.001	<0.001	<0.001	<0.001	<0.001	<0.001	<0.001

* Pearson’s Correlation Coefficient.

**Figure 4:**
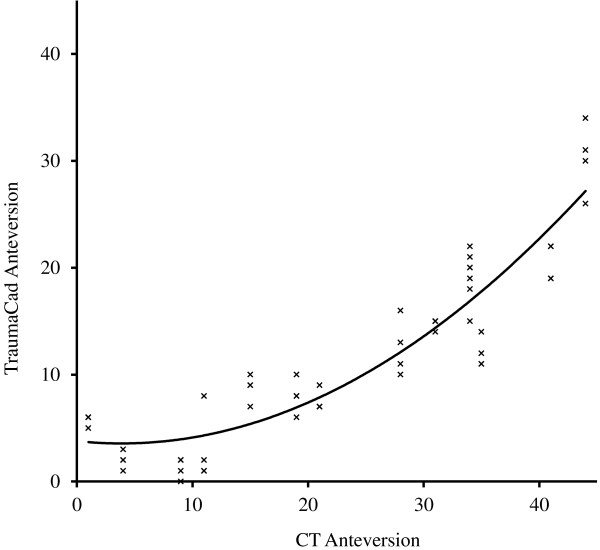
**Relationship between measurements of anteversion from CT and TraumaCad.** The data plots represent each observer’s first measurements with a line of best fit.

There was also strong correlation between the two methods for the measurement of version, although to a lesser degree than for inclination (Table 1). The mean absolute error was 12.0°. TraumaCad tended to underestimate the degree of version, as demonstrated by the flatter curve in Figure 4, although the relationship became more equal at higher degrees of version. Version was underestimated in 93% of the measurements taken, by an average of 12.6° (range 1° to 24°). When the average TraumaCad measurement was greater than 5°, adding 12° gave a reading within 5° of the CT measurement in 75% of cases.

All observers demonstrated good intra-observer reliability (Table 2) and there was good inter-observer reliability for each set of readings taken (Table 3).

**Table 2:** Intra-observer reliability of TraumaCad measurements

	**Observer 1**		**Observer 2**		**Observer 3**		**Observer 4**	
	ICC*	***p *value**	**ICC**	***p *value**	**ICC**	***p *value**	**ICC**	***p *value**
Inclination	0.992	<0.001	0.997	<0.001	0.992	<0.001	0.991	<0.001
Version	0.977	<0.001	0.989	<0.001	0.992	<0.001	0.901	<0.001

*intra-class correlation co-efficient (>0.7 = good correlation).

**Table 3:** Inter-observer reliability of TraumaCad measurements

	**1**^**st **^**readings**		**2**^**nd **^**readings**	
	ICC*	***p *value**	**ICC**	***p *value**
Inclination	0.971	<0.001	0.976	<0.001
Version	0.954	<0.001	0.962	<0.001

*intra-class correlation co-efficient (>0.7 = good correlation).

## Discussion

There is strong correlation between the two methods for the measurement of inclination and version, and good intra- and inter-observer reliability. However, TraumaCad underestimated version with respect to CT by around 12°.

There are a number of reasons why there should be inconsistency between the measurement of acetabular version from plain radiographs and CT. The profile of the component on a plain radiograph, and hence its apparent version, will be affected by the component’s position in three planes. There is likely to be a difference in pelvic tilt between the anatomical axis through which CT cuts are reconstructed and the axis in which the supine pelvis lies.

As recognised by Hart et al. [19], the small arc of the acetabular rim left uncovered by the large diameter femoral component makes accurate measurement of the ellipse difficult using this technique. The authors found that this was particularly difficult at the lower angles of version and indeed the measurement of version in this study correlated better with CT at higher degrees as seen in Figure 4.

**Figure 5:**
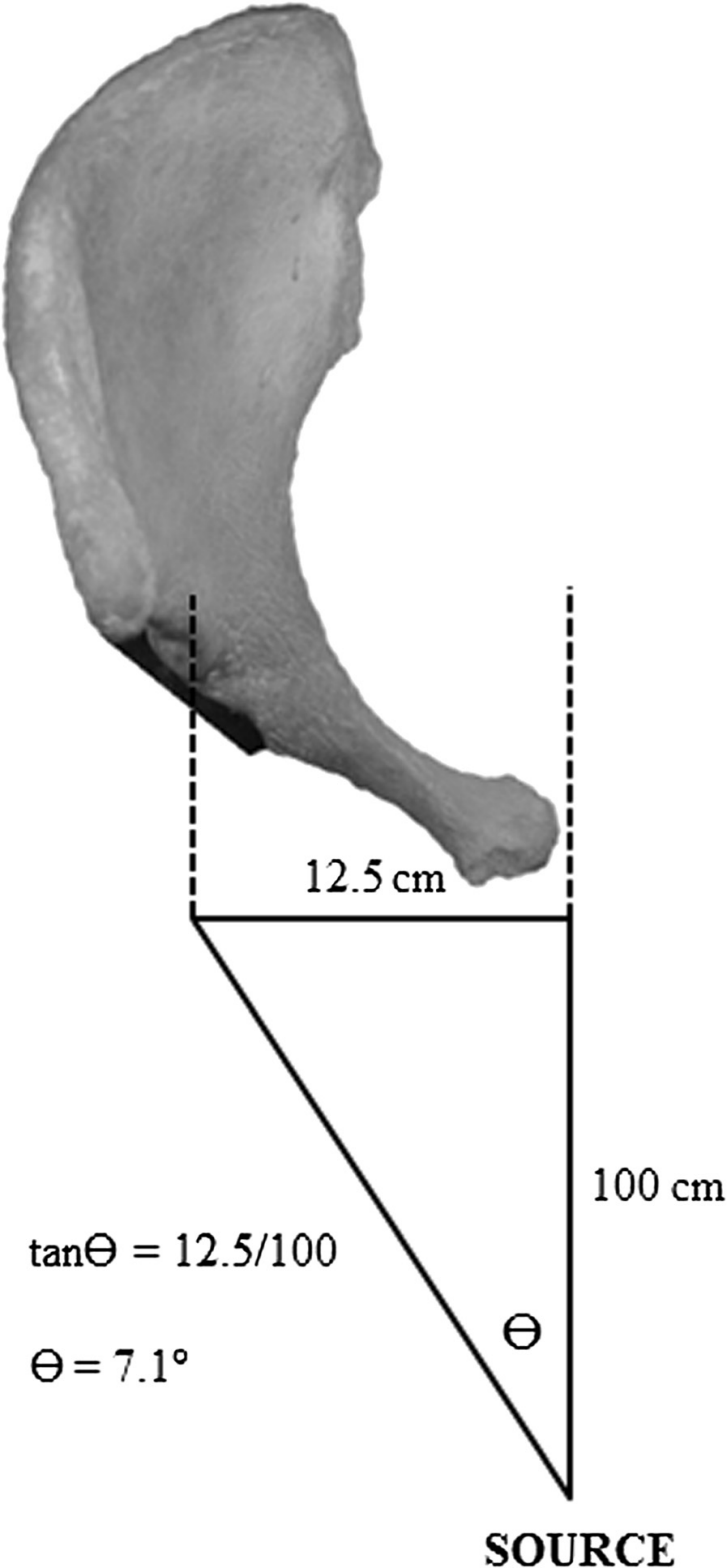
**Divergence of the X-Ray beam contributes to underestimation of version.** The distance between the femoral heads is assumed to be 25 cm and the X-Ray source is 100 cm from the patient.

Measurement of the angle of version may be underestimated due to the natural divergence of the X-Ray beam when a plain radiograph is taken and was described by Widmer [19]. Assuming a distance of 25 cm between the centres of the femoral heads, and assuming the X-Ray beam is focussed on the pubic symphysis from a distance of 100 cm, the beam will meet the acetabular component at an angle of 7.1° (tanӨ = opposite/adjacent), meaning a component that is anteverted by 7 would appear to be in neutral (Figure 5).

The measurement of a resurfacing cup may also be underestimated using this technique due to its non-hemispherical design. The ellipse technique relies on the S/TL ratio, as demonstrated in Figure 1. When using the TraumaCad software, in order to make the ellipse fit the articular border of the acetabular component and hence measure the version, the authors often found it necessary to have the line that should delineate the backside of the component sitting away from it due to the non-hemispherical design, thereby increasing TL in relation to S, and reducing the calculated version.

Although all the components in this study were in anteversion, it is important to remember that two-dimensional imaging cannot determine if a component is in anteversion or retroversion. The authors therefore include a lateral radiograph as part of their routine assessment. Although this method has been shown to lack accuracy [12], it is sufficient to demonstrate if a component is anteverted or retroverted.

EBRA software has been validated in measuring resurfacing cup anteversion [13]. It has the potential advantage of allowing the user to input bony landmarks to take into account any rotation or tilt of the pelvis. However, it is not as commonplace in orthopaedic departments in the UK as TraumaCad and departments may find it hard to justify the extra expenditure as financial constraints are tightened. This study suggests that the inability of TraumaCad to map bony landmarks is not a significant factor as we have demonstrated it to correlate with the CT reconstructions that measure orientation related to the bony anatomy of the pelvis.

As well as ultrasound, MRI and serum metal ion levels, the accurate assessment of the orientation of the acetabular component is vital in evaluating a patient with persistent groin pain following hip resurfacing arthroplasty, as edge-loading in excessively anteverted cups is thought to increase wear debris and subsequent adverse tissue reactions. This study has shown TraumaCad to have good intra- and inter-observer reliability, and to correlate well with CT, but the orthopaedic surgeon should expect the component to be some 12° more anteverted than measured. Armed with this knowledge, orthopaedic departments may choose not to invest in further expensive software such as EBRA, or to not expose their patients to the radiation exposure of CT.

It must be remembered that we are assuming CT to be a true representation of the orientation of the cup. CT scans are performed supine and therefore do not represent the functional position of the pelvis when walking upright. Pelvic obliquity due to leg length discrepancy or muscle imbalance and differences in pelvic tilt in the supine and standing positions will change the effective abduction angle and version when the patient stands. Since it is in the standing stance that the majority of loading occurs, it would seem more important to know the orientation of the cup relative to earth in this position when making an assessment of edge-loading. Although CT in a simulated-standing position is possible [17], standing radiographs are universally available. As well as saving radiation exposure and costs, the TraumaCad method therefore also confers the potential advantage of allowing assessment of acetabular orientation in the standing functional position. Although it underestimates to a degree, this inaccuracy may be compensated for by the benefits of assessing the functional position. There is an opportunity for further study investigating the relationship between component orientation measurement in supine and standing films.

## Conclusions

Following hip resurfacing with a non-hemispherical component, measurement of acetabular orientation using TraumaCad correlates well with CT and has good intra- and inter-observe reliability. It can however be expected to underestimate version by around 12°. Remembering this, when assessing a patient with persistent pain following hip resurfacing, the orthopaedic surgeon can use TraumaCad in the place of CT, or other software such as EBRA, which may reduce radiation exposure and costs.

## Competing interests

The authors declare that they have no competing interests.

## Authors’ contributions

All authors performed measurements as detailed in the Methods section. DW wrote the paper. JM helped conceive the study. RK performed the statistical analysis. PF identified the study population and supervised the study. All authors read and approved the final manuscript.
